# (2*Z*)-3-Hy­droxy-1-(pyridin-2-yl)-3-(pyridin-3-yl)prop-2-en-1-one: crystal structure and Hirshfeld surface analysis

**DOI:** 10.1107/S205698901600832X

**Published:** 2016-05-27

**Authors:** Sze-Ling Lee, Ai Ling Tan, David J. Young, Mukesh M. Jotani, Edward R. T. Tiekink

**Affiliations:** a Faculty of Science, Universiti Brunei Darussalam, Jalan Tungku Link BE 1410, Negara Brunei Darussalam; b Faculty of Science, Health, Education and Engineering, University of the Sunshine Coast, Maroochydore DC, Queensland 4558, Australia; c Department of Physics, Bhavan’s Sheth R. A. College of Science, Ahmedabad, Gujarat 380 001, India; d Research Centre for Crystalline Materials, Faculty of Science and Technology, Sunway University, 47500 Bandar Sunway, Selangor Darul Ehsan, Malaysia

**Keywords:** crystal structure, propane-1,3-dione, hydrogen bond, Hirshfeld surface

## Abstract

The title compound, C_13_H_10_N_2_O_2_ [also called 1-(pyridin-2-yl)-3-(pyridin-3-yl)propane-1,3-dione], features an almost planar (r.m.s. deviation = 0.0095 Å) central C_3_O_2_ core consolidated by an intra­molecular hy­droxy-O—H⋯O(carbon­yl) hydrogen bond. Twists are evident in the mol­ecule, as seen in the dihedral angles between the central core and the 2- and pyridin-3-yl rings of 8.91 (7) and 15.88 (6)°, respectively. The conformation about the C=C bond [1.3931 (17) Å] is *Z*, and the N atoms lie to the same side of the mol­ecule. In the mol­ecular packing, supra­molecular chains along the *a* axis are mediated by π(pyridin-2-yl)–π(pyridin-3-yl) inter­actions [inter-centroid distance = 3.7662 (9) Å]. The observation that chains pack with no directional inter­actions between them is consistent with the calculated electrostatic potential, which indicates that repulsive inter­actions dominate.

## Abstract 2

The title mol­ecule, featuring an intra­molecular O—H⋯O hydrogen bond, is non-planar as seen in the dihedral angle between the pyridyl rings of 7.45 (7)°. In the crystal, supra­molecular chains are formed *via* π(pyridin-2-yl)–π(pyridin-3-yl) inter­actions.

## Chemical context   

The β-diketonates of virtually all metals are known (Lamprey, 1960 ▸) because of the stability of the resulting six-membered metallocycle formed from bidentate coordination through the two oxygen atoms and the ability of the ligand to be accommodated within the common octa­hedral, tetra­hedral and square-pyramidal coordination geometries. There has been an inter­est over the last few years to introduce extra donor functionality such as nitrile and pyridyl to this class of ligand to generate heterometallic complexes and novel coordination networks. Dipyridyl β-diketonates, for example, have been used to synthesize mixed-metal–organic frameworks. Burrows and co-workers employed di(pyridin-4-yl)propane-1,3-dione to prepare the corresponding Al^III^ and Ga^III^ octa­hedral building blocks for network structures linked by Ag^I^ ions (Burrows *et al.*, 2010 ▸). Carlucci and co-workers used the same ligand to make Fe^III^ metalloligands that were again joined by coordination to Ag^I^ ions. The type of the resulting two- or three-dimensional coordination polymer depended on the nature of the counter-ion to silver (Carlucci *et al.*, 2011 ▸). By comparison, the di(pyridin-2-yl)propane-1,3-dione ligand, which also has extra donor functionality available for coord­ination, is sterically hindered to allow network formation. Tan and co-workers prepared the Cd^II^ and Cu^II^ complexes from this ligand and did indeed observe chelation through the 2,2′-nitro­gen atoms (Tan *et al.*, 2012 ▸). However, they did not observe solid-state network formation from bridging oxygen-atom, μ_2_-Cl or μ_3_-Cl donors in the Cd^II^ complexes; the Cu^II^ complex was a tetra­nuclear oligomer linked *via* bridging water and acetate counter-ions (Tan *et al.*, 2012 ▸). Less work has been performed with the unsymmetrical pyridyl β-diketonates. Zhang and co-workers have made the Fe^III^ salt of 3-(pyridin-4-yl)-2,4-penta­nedione as well as the mixed-MOF with AgNO_3_ in a two-dimensional honeycomb structure while at higher Ag^I^ concentrations, a one-dimensional ladder motif was formed (Zhang *et al.*, 2008 ▸). This ligand and the symmetrical 4,4′- and 3,3′- variants have been treated with hydrazine to give the corresponding pyrazoles that were used to prepare strongly photoluminescent Cu^I^ coordination polymers (Zhan *et al.*, 2011 ▸).
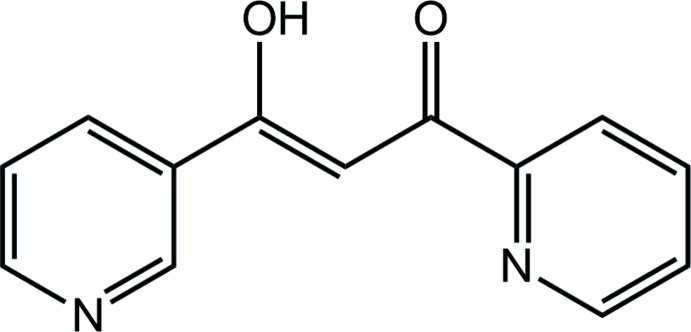


All of the mentioned dipyridyl ligands can be conveniently prepared by the Claisen condensation of an acetyl­pyridine with a pyridine carb­oxy­lic ester. The title compound, (I), has not previously been reported, but was prepared in this way from 2-acetyl­pyridine and ethyl nicotinate, and crystals suitable for X-ray crystallography were obtained by recrystallization from a mixture of di­chloro­methane and hexane. Herein, the crystal structure analysis of (I) is described along with a detailed investigation of the mol­ecular packing by a Hirshfeld surface analysis.

## Structural commentary   

**Figure 1:**
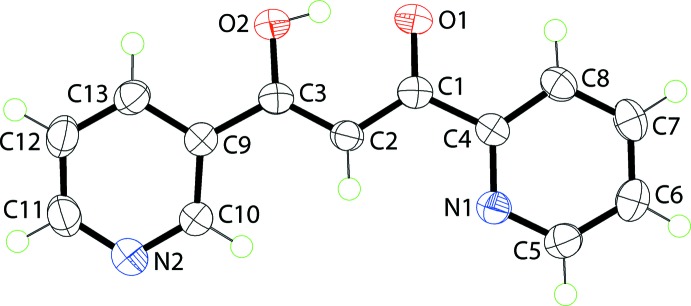
The mol­ecular structure of (I), showing the atom-labelling scheme and displacement ellipsoids at the 70% probability level.

In (I), the assignment of carbonyl- *versus* hy­droxy-O atoms is not readily confirmed by a great disparity in the C1—O1 [1.2871 (14) Å] and C3—O2 [1.3041 (14) Å] bond lengths. The assignment was based on an unrestrained refinement of the H1*O* atom which resulted in a O2—H1*O* bond length of 1.090 (18) Å. More certainty is associated with the assignment of the nitro­gen atoms in the pyridyl rings. Thus, the short C5—N1 and C4—N1 [1.3325 (17) and 1.3484 (15) Å] and C10—N2 and C11—N2 [1.3371 (17) and 1.3397 (18) Å] bond lengths *cf*. the C—C bonds in the rings confirm their assignment. The central C_3_O_2_ residual in (I), Fig. 1 ▸, is essentially planar with the r.m.s. deviation of the five atoms being 0.0095 Å. The *syn* arrangement of the oxygen atoms enables the formation of an intra­molecular hy­droxy-O—H⋯O(carbon­yl) hydrogen bond, Table 1 ▸. The dihedral angles formed between the central plane and the N1- and N2-pyridinyl rings are 8.91 (7) and 15.88 (6)°, respectively, indicating twists in the mol­ecule. The dihedral angle between the pyridyl rings is 7.45 (7)°. The conformation about the C2=C3 [1.3931 (17) Å] is *Z*, and, to a first approximation, the N1 and N2 atoms lie to the same side of the mol­ecule.

## Supra­molecular features   

**Figure 2:**
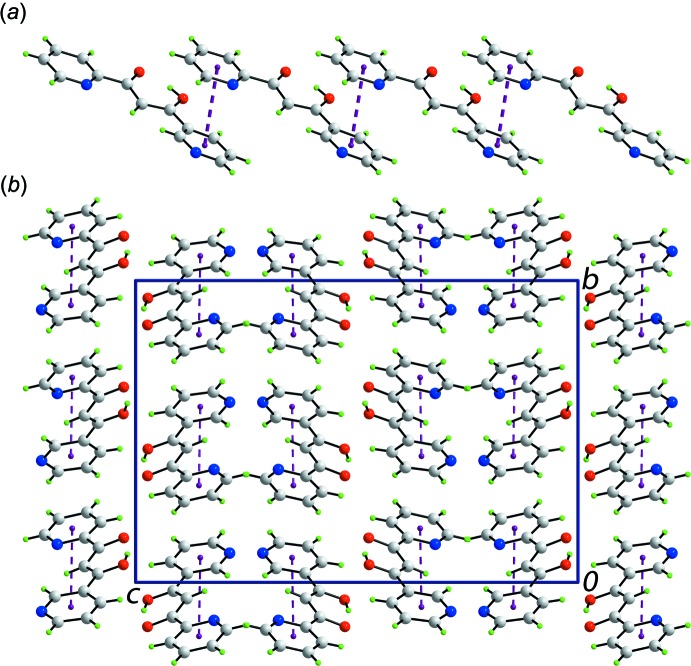
Mol­ecular packing in (I): (*a*) a view of the supra­molecular chain along the *a* axis sustained by π–π inter­actions and (*b*) unit-cell contents shown in projection down the *a* axis. The π–π inter­actions are shown as purple dashed lines.

The mol­ecular packing in the crystal is dominated by π–π inter­actions formed between the N1- and N2-pyridinyl rings of translationally related mol­ecules [*Cg*(N1-pyridin­yl)⋯*Cg*(N2-pyridin­yl) = 3.7662 (9) Å, angle of inclination = 7.45 (6)° for symmetry operation 1 + *x*, *y*, *z*]. The result is the formation of a linear supra­molecular chain, Fig. 2 ▸ *a*. The chains pack with no directional inter­actions between them in accord with the distance criteria in *PLATON* (Spek, 2009 ▸), Fig. 2 ▸ *b*.

## Hirshfeld surface analysis   

The program *Crystal Explorer 3.1* (Wolff *et al.*, 2012 ▸) was used to generate Hirshfeld surfaces mapped over the electrostatic potential, *d*_norm_, shape-index and curvedness. The electrostatic potential was calculated with *TONTO* (Spackman *et al.*, 2008 ▸; Jayatilaka *et al.*, 2005 ▸), integrated in *Crystal Explorer*, using the experimental geometry as the input. The electrostatic potentials were mapped on the Hirshfeld surface using the STO-3G basis set at the Hartree–Fock level of theory over a range ±0.06 au. The contact distances *d*_i_ and *d*_e_ from the Hirshfeld surface to the nearest atom inside and outside, respectively, enables the analysis of the inter­molecular inter­actions through the mapping of *d*_norm_. The combination of *d*_e_ and *d*_i_ in the form of a two-dimensional fingerprint plot (McKinnon *et al.*, 2004 ▸) provides a summary of the inter­molecular contacts in the crystal.

**Figure 3:**
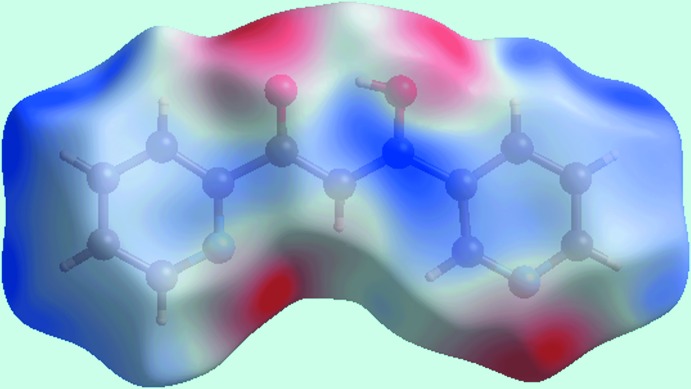
A view of the Hirshfeld surface mapped over electrostatic potential for (I). The red and blue regions represent negative and positive electrostatic potentials, respectively.

**Figure 4:**
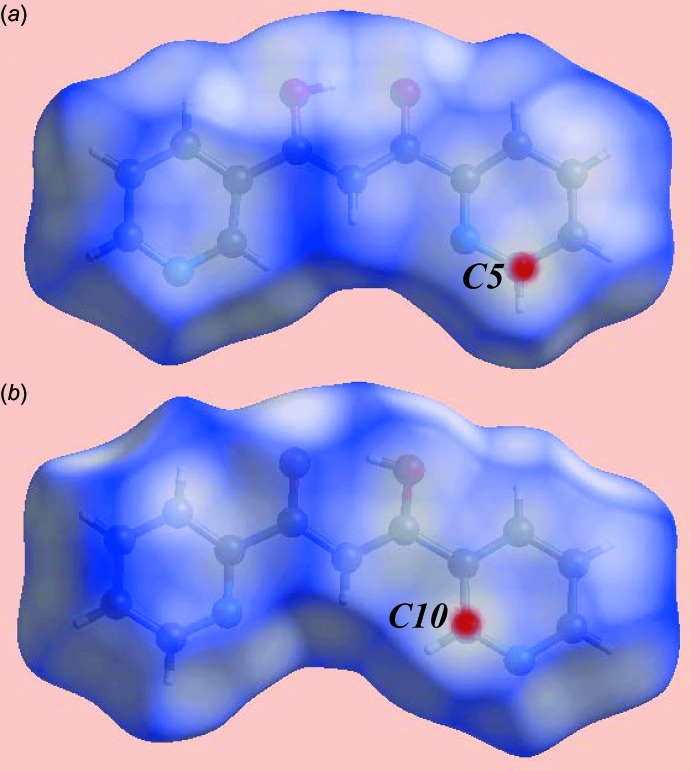
Two views of the Hirshfeld surface mapped over *d*_norm_ for (I): the bright-red spots at (*a*) C5 and (*b*) C10 indicate their involvement in short inter­molecular C⋯C contacts.

From the Hirshfeld surface mapped over electrostatic potential, Fig. 3 ▸, the negative potentials around the oxygen atoms of the hy­droxy and carbonyl groups as well as about the nitro­gen atoms of pyridyl rings prevent their participation in inter­molecular inter­actions in the crystal of (I) due to the electrostatic repulsion that would eventuate. The presence of a short inter­molecular C⋯C contact between the C5 and C10 atoms [C5⋯C10 = 3.313 (2) Å; symmetry code: −1 + *x*, *y*, *z*], which fall within the π–π contacts between pyridyl rings (Fig. 2 ▸ *a*), is viewed as bright-red spots near these atoms on the Hirshfeld surface mapped over *d*_norm_, Fig. 4 ▸.

**Figure 5:**
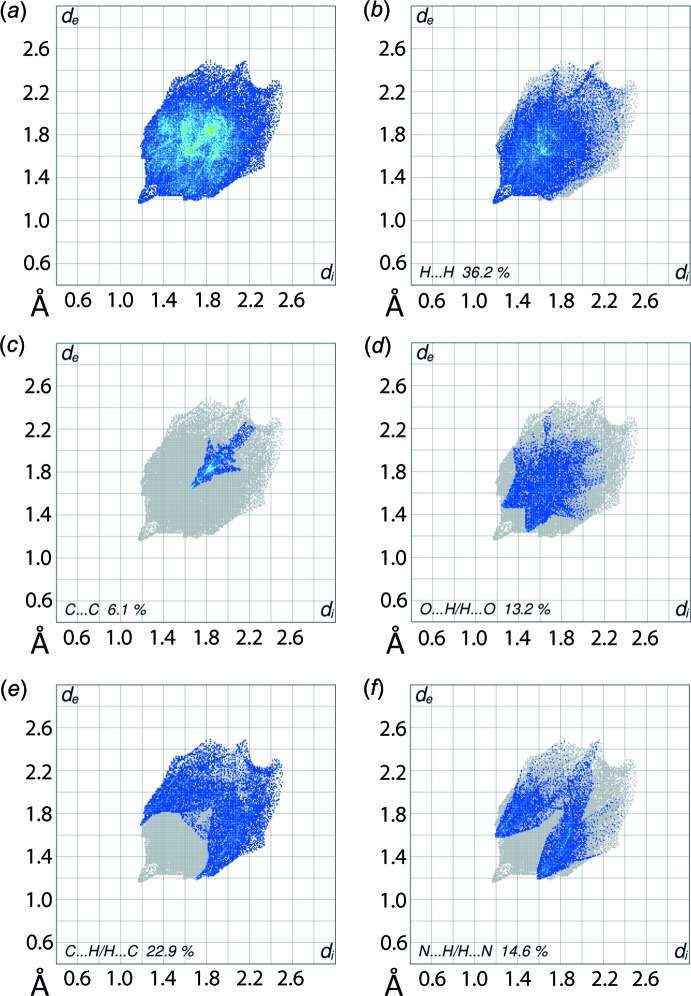
The two-dimensional fingerprint plots for (I): (*a*) all inter­actions, and delineated into (*b*) H⋯H, (*c*) C⋯C, (*d*) O⋯H/H⋯O, (*e*) C⋯H/H⋯C and (*f*) N⋯H/H⋯N inter­actions.

**Figure 6:**
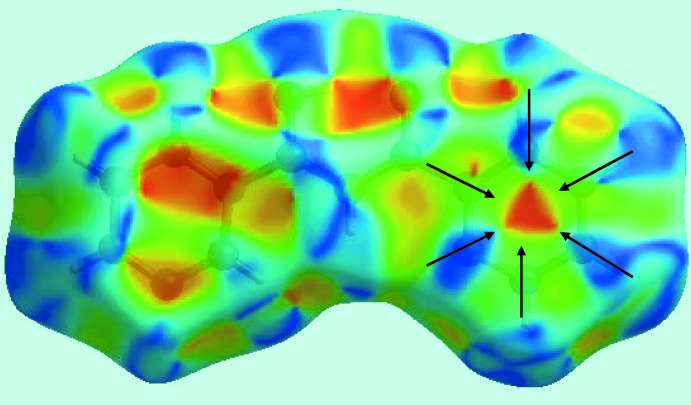
A view of the Hirshfeld surface mapped with shape-index property for (I). The red and blue triangles identified with arrows indicate π–π stacking inter­actions.

**Figure 7:**
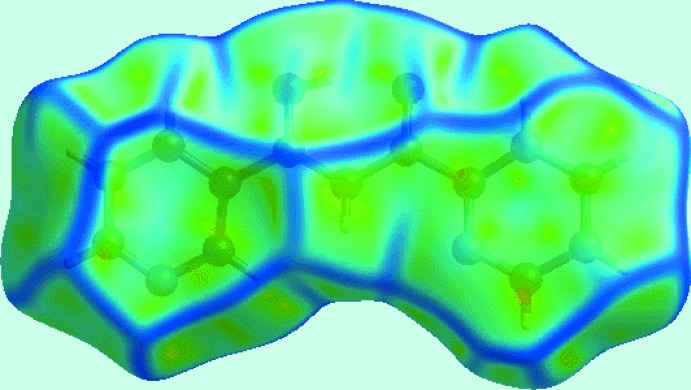
A view of the Hirshfeld surface mapped over curvedness for (I). The flat regions highlight the involvement of rings in the π–π stacking inter­actions.

The overall 2D fingerprint plot, Fig. 5 ▸ *a*, and those delin­eated into H⋯H, C⋯C, O⋯H/H⋯O, C⋯H/H⋯C and N⋯H/H⋯N contacts are illustrated in Fig. 5 ▸ *b*–*f*, respectively; their relative contributions to the surface are qu­anti­fied in Table 2 ▸. The inter­atomic H⋯H contacts (McKinnon *et al.*, 2007 ▸) appear as the scattered points over the greater part of the plot shown in Fig. 5 ▸ *b*, with a single peak at (*d*_e_, *d*_i_) less than the van der Waals separation corresponding to a short H13⋯H13 contact of 2.33 Å (symmetry code: 1 − *x*, −*y*, −*z*). The short inter­atomic C5⋯C10 contact and π–π stacking inter­actions appear as an arrow-like distribution of points with the tip at *d*_e_ + *d*_i_ ∼ 3.3 Å (Fig. 5 ▸ *c*). The presence of π–π stacking inter­actions between the pyridyl rings is also apparent from the appearance of red and blue triangle pairs on the Hirshfeld surface mapped with shape-index property identified with arrows in the image of Fig. 6 ▸, and in the flat region on the Hirshfeld surface mapped over curvedness in Fig. 7 ▸.

The two-dimensional fingerprint plots delineated into O⋯H/H⋯O, C⋯H/H⋯C and N⋯H/H⋯N inter­actions exhibit their usual characteristic features in their respective plots; Fig. 4 ▸ *d*–*f*. However, the points are distributed at (*d*_e_, *d*_i_) distances greater than their respective van der Waals separations. This is consistent with the repulsion between the atoms having electrostatic negative potential dominating the mol­ecular packing, hence the lack of specific inter­molecular inter­actions between supra­molecular chains.

## Database survey   

**Figure 8:**
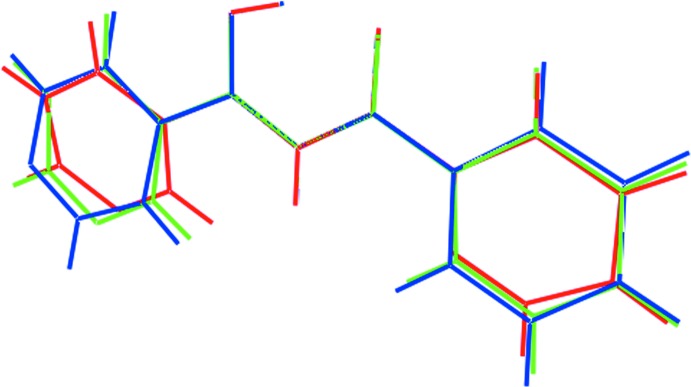
Overlay diagram of mol­ecules of (I) (red image), (II) (green) and (III) (blue). The mol­ecules have been overlapped so that the central five-membered rings are coincident.

A survey of the Cambridge Structural Database (Groom *et al.*, 2016 ▸) revealed that there are two closely related pyridyl-substituted propane-1,3-dione structures in the crystallographic literature. These are the mono-pyridyl derivatives 3-hy­droxy-1-phenyl-3-(pyridin-3-yl)prop-2-en-1-one (II) and 3-hy­droxy-1-phenyl-3-(pyridin-4-yl)prop-2-en-1-one (III), both published by Dudek *et al.* (2011 ▸). Each structure features a very similar central core with the intra­molecular O—H⋯O hydrogen bond. In each of (II) and (III), the pyridyl ring is connected to the carbon atom bearing the hy­droxy group. As seen from the overlay diagram (Fig. 8 ▸) and as qu­anti­fied in Table 3 ▸, the three structures (I)–(III) have very similar conformations.

## Synthesis and crystallization   

2-Acetyl­pyridine (3.0562 g, 25.2 mmol) was added to a suspension of NaH (60% dispersion in mineral oil, 2.0058 g, 50.0 mmol) in anhydrous THF (10 ml) at room temperature with stirring. Ethyl nicotinate (7.5675g, 50.1 mmol) in anhydrous THF (10ml) was added dropwise to the mixture over 3 min. The yellow mixture was refluxed under a nitro­gen atmosphere for 1.3 h and then quenched with ice–water (50 ml). Glacial acetic acid was added to adjust the pH to 6–7. The resulting yellow precipitate was collected by filtration, washed with cold water and dried under vacuum. Recrystallization from di­chloro­methane–hexane (1:1 *v*/*v*) solution afforded colourless crystals. Yield: 4.03 g (70.7%). M.p: 377–378 K. IR (KBr pellet) ν_max_/cm^−1^: 3121 (*m*), 3053 (*m*), 2922 (*m*), 2853 (*m*), 1611 (*s*), 1595 (*s*), 1539 (*m*), 1458 (*m*), 1418 (*m*), 1221 (*m*), 1188 (*m*), 1146 (*m*), 1115 (*m*), 1067 (*m*), 1018 (*m*), 989 (*m*), 926 (*m*), 775 (*s*), 739 (*m*), 679 (*s*), 611 (*m*). Analysis calculated for C_13_H_10_N_2_O_2_: C, 69.03; H, 4.42; N, 12.19. Found: C, 68.73; H, 4.54; N, 12.16. MS: *m*/*z* 226. ^1^H NMR (400 MHz, *d*_6_–DMSO) δ 9.22 (1H, *s*), 8.82 (2H, *m*), 8.44 (1H, *d*, *J* = 7.9 Hz), 8.17 (1H, *d*, *J* = 7.8 Hz), 8.09 (1H, *m*), 7.70 (1H, *m*), 7.63 (2H, *m*).

## Refinement details   

Crystal data, data collection and structure refinement details are summarized in Table 4 ▸. Carbon-bound H atoms were placed in their calculated positions (C—H = 0.95 Å) and were included in the refinement in the riding-model approximation, with *U*_iso_(H) set to 1.2*U*_eq_(C). The hy­droxy-H atom was located in a difference map and refined with O—H = 0.82±0.01Å, and with *U*_iso_(H) set to 1.5*U*_eq_(O).

## Supplementary Material

Crystal structure: contains datablock(s) I, global. DOI: 10.1107/S205698901600832X/hb7587sup1.cif

Structure factors: contains datablock(s) I. DOI: 10.1107/S205698901600832X/hb7587Isup2.hkl

Supporting information file. DOI: 10.1107/S205698901600832X/hb7587Isup3.cml

CCDC reference: 1481225

Additional supporting information:  crystallographic information; 3D view; checkCIF report

## Figures and Tables

**Table 1:** Hydrogen-bond geometry (Å, °)

*D*—H⋯*A*	*D*—H	H⋯*A*	*D*⋯*A*	*D*—H⋯*A*
O2—H2*O*⋯O1	0.85 (2)	1.65 (1)	2.4673 (14)	160 (2)

**Table 2:** Percentage contribution of the different inter­molecular inter­actions to the Hirshfeld surface of (I)

Contact	%
H⋯H	36.2
O⋯H/H⋯O	13.2
C⋯H/H⋯C	22.9
N⋯H/H⋯N	14.6
C⋯C	6.1
C⋯O/O⋯C	2.9
C⋯N/N⋯C	2.8
O⋯O	0.9
N⋯N	0.4

**Table 3:** Dihedral angle (°) data for (I)–(III)

Structure	C_3_O_2_/*n*-pyrid­yl	C_3_O_2_/pyridin-2-yl or phen­yl	ring/ring	CSD refcode^*a*^	Reference
(I)	*n* = 3; 15.88 (6)	8.91 (7)	7.45 (7)	–	This work
(II)	*n* = 3; 2.23 (9)	4.20 (8)	4.38 (9)	XIOXID	Dudek *et al.* (2011 ▸)
(III)^*b*^	*n* = 4; 8.10 (5)	11.41 (5)	3.88 (5)	BEDREJ	Dudek *et al.* (2011 ▸)

Notes: (*a*) Groom *et al.* (2016 ▸); (*b*) isolated as a 1:1 co-crystal with benzoic acid.

**Table 4:** Experimental details

Crystal data	
Chemical formula	C_13_H_10_N_2_O_2_
*M*_r_	226.23
Crystal system, space group	Orthorhombic, *P* *b* *c* *a*
Temperature (K)	273
*a*, *b*, *c* (Å)	7.2124 (9), 14.1782 (19), 20.794 (3)
*V* (Å^3^)	2126.4 (5)
*Z*	8
Radiation type	Mo *K*α
μ (mm^−1^)	0.10
Crystal size (mm)	0.20 × 0.20 × 0.19

Data collection	
Diffractometer	Bruker D8-Quest CCD
Absorption correction	Multi-scan (*SADABS*; Sheldrick, 1996 ▸)
*T*_min_, *T*_max_	0.981, 0.982
No. of measured, independent and observed [*I* > 2σ(*I*)] reflections	46632, 2647, 2227
*R*_int_	0.057
(sin θ/λ)_max_ (Å^−1^)	0.671

Refinement	
*R*[*F* ^2^ > 2σ(*F* ^2^)], *wR*(*F* ^2^), *S*	0.042, 0.121, 1.05
No. of reflections	2647
No. of parameters	157
No. of restraints	1
Δρ_max_, Δρ_min_ (e Å^−3^)	0.33, −0.24

Computer programs: *SMART* and *SAINT* (Bruker, 2007 ▸), *SHELXS97* (Sheldrick, 2008 ▸), *SHELXL2014* (Sheldrick, 2015 ▸), *ORTEP-3 for Windows* (Farrugia, 2012 ▸), *QMol* (Gans & Shalloway, 2001 ▸), *DIAMOND* (Brandenburg, 2006 ▸) and *publCIF* (Westrip, 2010 ▸).

## supplementary crystallographic information

### Crystal data

**Table:**

C_13_H_10_N_2_O_2_	*D*_x_ = 1.413 Mg m^−^^3^
*M**_r_* = 226.23	Mo *K*α radiation, λ = 0.71073 Å
Orthorhombic, *P**b**c**a*	Cell parameters from 9904 reflections
*a* = 7.2124 (9) Å	θ = 3.0–28.3°
*b* = 14.1782 (19) Å	µ = 0.10 mm^−^^1^
*c* = 20.794 (3) Å	*T* = 273 K
*V* = 2126.4 (5) Å^3^	Block, colourless
*Z* = 8	0.20 × 0.20 × 0.19 mm
*F*(000) = 944	

### Data collection

**Table:**

Bruker D8-Quest CCD diffractometer	2647 independent reflections
Radiation source: sealed tube	2227 reflections with *I* > 2σ(*I*)
Graphite monochromator	*R*_int_ = 0.057
Detector resolution: 8.366 pixels mm^-1^	θ_max_ = 28.5°, θ_min_ = 3.0°
φ and ω scans	*h* = −8→9
Absorption correction: multi-scan (*SADABS*; Sheldrick, 1996)	*k* = −18→18
*T*_min_ = 0.981, *T*_max_ = 0.982	*l* = −27→27
46632 measured reflections	

### Refinement

**Table:**

Refinement on *F*^2^	1 restraint
Least-squares matrix: full	Hydrogen site location: mixed
*R*[*F*^2^ > 2σ(*F*^2^)] = 0.042	*w* = 1/[σ^2^(*F*_o_^2^) + (0.0588*P*)^2^ + 0.9349*P*] where *P* = (*F*_o_^2^ + 2*F*_c_^2^)/3
*wR*(*F*^2^) = 0.121	(Δ/σ)_max_ < 0.001
*S* = 1.05	Δρ_max_ = 0.33 e Å^−^^3^
2647 reflections	Δρ_min_ = −0.24 e Å^−^^3^
157 parameters	

### Special details

**Table:**

Geometry. All esds (except the esd in the dihedral angle between two l.s. planes) are estimated using the full covariance matrix. The cell esds are taken into account individually in the estimation of esds in distances, angles and torsion angles; correlations between esds in cell parameters are only used when they are defined by crystal symmetry. An approximate (isotropic) treatment of cell esds is used for estimating esds involving l.s. planes.

### Fractional atomic coordinates and isotropic or equivalent isotropic displacement parameters (Å^2^)

**Table:**

	*x*	*y*	*z*	*U*_iso_*/*U*_eq_	
O1	−0.09862 (13)	0.14710 (7)	0.02692 (4)	0.0286 (2)	
O2	0.19541 (13)	0.05816 (7)	0.02776 (4)	0.0276 (2)	
H2O	0.1025 (19)	0.0929 (11)	0.0188 (8)	0.041*	
N1	−0.33075 (15)	0.12603 (7)	0.17723 (5)	0.0246 (2)	
N2	0.43366 (17)	−0.09680 (9)	0.21246 (6)	0.0335 (3)	
C1	−0.13288 (17)	0.11505 (8)	0.08360 (6)	0.0217 (3)	
C2	−0.01053 (16)	0.05315 (8)	0.11536 (5)	0.0214 (3)	
H2	−0.0402	0.0290	0.1557	0.026*	
C3	0.15550 (17)	0.02859 (8)	0.08551 (5)	0.0211 (2)	
C4	−0.30743 (16)	0.14778 (8)	0.11462 (5)	0.0203 (2)	
C5	−0.48525 (19)	0.15721 (9)	0.20520 (6)	0.0286 (3)	
H5	−0.5044	0.1425	0.2483	0.034*	
C6	−0.61954 (19)	0.21046 (9)	0.17384 (7)	0.0294 (3)	
H6	−0.7244	0.2313	0.1957	0.035*	
C7	−0.59415 (18)	0.23185 (9)	0.10951 (7)	0.0284 (3)	
H7	−0.6819	0.2669	0.0871	0.034*	
C8	−0.43511 (18)	0.19984 (8)	0.07917 (6)	0.0245 (3)	
H8	−0.4141	0.2129	0.0360	0.029*	
C9	0.29989 (16)	−0.03006 (8)	0.11646 (5)	0.0206 (2)	
C10	0.30159 (18)	−0.04710 (9)	0.18265 (6)	0.0269 (3)	
H10	0.2055	−0.0225	0.2073	0.032*	
C11	0.57090 (19)	−0.13133 (10)	0.17598 (7)	0.0315 (3)	
H11	0.6644	−0.1656	0.1960	0.038*	
C12	0.58095 (19)	−0.11875 (9)	0.10999 (7)	0.0308 (3)	
H12	0.6782	−0.1445	0.0865	0.037*	
C13	0.44368 (18)	−0.06720 (9)	0.07980 (6)	0.0257 (3)	
H13	0.4474	−0.0575	0.0356	0.031*	

### Atomic displacement parameters (Å^2^)

**Table:**

	*U*^11^	*U*^22^	*U*^33^	*U*^12^	*U*^13^	*U*^23^
O1	0.0318 (5)	0.0325 (5)	0.0214 (4)	0.0031 (4)	0.0021 (3)	0.0078 (3)
O2	0.0272 (5)	0.0349 (5)	0.0208 (4)	0.0036 (4)	0.0036 (3)	0.0050 (3)
N1	0.0235 (5)	0.0280 (5)	0.0224 (5)	−0.0003 (4)	−0.0005 (4)	0.0020 (4)
N2	0.0316 (6)	0.0394 (6)	0.0294 (6)	0.0077 (5)	−0.0005 (5)	0.0056 (5)
C1	0.0229 (6)	0.0215 (5)	0.0207 (5)	−0.0035 (4)	−0.0010 (4)	−0.0003 (4)
C2	0.0222 (6)	0.0232 (6)	0.0188 (5)	−0.0003 (4)	−0.0006 (4)	0.0020 (4)
C3	0.0226 (6)	0.0211 (5)	0.0195 (5)	−0.0033 (4)	−0.0009 (4)	−0.0010 (4)
C4	0.0225 (6)	0.0173 (5)	0.0211 (5)	−0.0022 (4)	−0.0023 (4)	−0.0002 (4)
C5	0.0282 (6)	0.0333 (7)	0.0243 (6)	0.0001 (5)	0.0023 (5)	0.0003 (5)
C6	0.0250 (6)	0.0277 (6)	0.0355 (7)	0.0030 (5)	0.0034 (5)	−0.0046 (5)
C7	0.0275 (6)	0.0220 (6)	0.0357 (7)	0.0049 (5)	−0.0053 (5)	0.0007 (5)
C8	0.0286 (6)	0.0209 (5)	0.0240 (6)	0.0006 (5)	−0.0034 (5)	0.0013 (4)
C9	0.0202 (5)	0.0191 (5)	0.0224 (5)	−0.0030 (4)	−0.0001 (4)	−0.0010 (4)
C10	0.0247 (6)	0.0320 (6)	0.0239 (6)	0.0044 (5)	0.0020 (5)	0.0022 (5)
C11	0.0263 (7)	0.0309 (7)	0.0373 (7)	0.0055 (5)	−0.0021 (5)	0.0043 (5)
C12	0.0276 (6)	0.0279 (6)	0.0369 (7)	0.0066 (5)	0.0059 (5)	−0.0008 (5)
C13	0.0284 (6)	0.0245 (6)	0.0243 (6)	0.0005 (5)	0.0036 (5)	−0.0012 (5)

### Geometric parameters (Å, º)

**Table:**

O1—C1	1.2871 (14)	C5—H5	0.9300
O2—C3	1.3041 (14)	C6—C7	1.3839 (19)
O2—H2O	0.853 (9)	C6—H6	0.9300
N1—C5	1.3325 (17)	C7—C8	1.3855 (18)
N1—C4	1.3484 (15)	C7—H7	0.9300
N2—C10	1.3371 (17)	C8—H8	0.9300
N2—C11	1.3397 (18)	C9—C13	1.3907 (17)
C1—C2	1.4090 (16)	C9—C10	1.3975 (16)
C1—C4	1.4888 (17)	C10—H10	0.9300
C2—C3	1.3931 (17)	C11—C12	1.386 (2)
C2—H2	0.9300	C11—H11	0.9300
C3—C9	1.4800 (16)	C12—C13	1.3815 (18)
C4—C8	1.3915 (16)	C12—H12	0.9300
C5—C6	1.3905 (19)	C13—H13	0.9300
			
C3—O2—H2O	102.4 (12)	C6—C7—C8	118.55 (12)
C5—N1—C4	116.73 (11)	C6—C7—H7	120.7
C10—N2—C11	117.16 (12)	C8—C7—H7	120.7
O1—C1—C2	121.93 (11)	C7—C8—C4	118.71 (11)
O1—C1—C4	116.69 (10)	C7—C8—H8	120.6
C2—C1—C4	121.37 (10)	C4—C8—H8	120.6
C3—C2—C1	119.02 (11)	C13—C9—C10	117.90 (11)
C3—C2—H2	120.5	C13—C9—C3	119.94 (11)
C1—C2—H2	120.5	C10—C9—C3	122.12 (11)
O2—C3—C2	121.32 (11)	N2—C10—C9	123.63 (12)
O2—C3—C9	115.20 (10)	N2—C10—H10	118.2
C2—C3—C9	123.47 (10)	C9—C10—H10	118.2
N1—C4—C8	123.38 (11)	N2—C11—C12	123.53 (12)
N1—C4—C1	116.91 (10)	N2—C11—H11	118.2
C8—C4—C1	119.69 (11)	C12—C11—H11	118.2
N1—C5—C6	123.92 (12)	C13—C12—C11	118.73 (12)
N1—C5—H5	118.0	C13—C12—H12	120.6
C6—C5—H5	118.0	C11—C12—H12	120.6
C7—C6—C5	118.69 (12)	C12—C13—C9	119.05 (12)
C7—C6—H6	120.7	C12—C13—H13	120.5
C5—C6—H6	120.7	C9—C13—H13	120.5
			
O1—C1—C2—C3	2.34 (18)	N1—C4—C8—C7	0.49 (18)
C4—C1—C2—C3	−176.70 (10)	C1—C4—C8—C7	−178.19 (11)
C1—C2—C3—O2	−3.26 (17)	O2—C3—C9—C13	−14.19 (16)
C1—C2—C3—C9	175.33 (10)	C2—C3—C9—C13	167.14 (11)
C5—N1—C4—C8	−0.14 (17)	O2—C3—C9—C10	163.35 (11)
C5—N1—C4—C1	178.58 (11)	C2—C3—C9—C10	−15.33 (18)
O1—C1—C4—N1	−170.37 (10)	C11—N2—C10—C9	0.2 (2)
C2—C1—C4—N1	8.72 (17)	C13—C9—C10—N2	0.14 (19)
O1—C1—C4—C8	8.40 (16)	C3—C9—C10—N2	−177.44 (12)
C2—C1—C4—C8	−172.51 (10)	C10—N2—C11—C12	−0.6 (2)
C4—N1—C5—C6	−0.55 (19)	N2—C11—C12—C13	0.6 (2)
N1—C5—C6—C7	0.9 (2)	C11—C12—C13—C9	−0.27 (19)
C5—C6—C7—C8	−0.48 (19)	C10—C9—C13—C12	−0.09 (18)
C6—C7—C8—C4	−0.16 (18)	C3—C9—C13—C12	177.55 (11)

### Hydrogen-bond geometry (Å, º)

**Table:**

*D*—H···*A*	*D*—H	H···*A*	*D*···*A*	*D*—H···*A*
O2—H2*O*···O1	0.85 (2)	1.65 (1)	2.4673 (14)	160 (2)

## References

[bb1] Brandenburg, K. (2006). *DIAMOND*. Crystal Impact GbR, Bonn, Germany.

[bb2] Bruker (2007). *SMART* and *SAINT*. Bruker AXS Inc., Madison, Wisconsin, USA.

[bb3] Burrows, A. D., Frost, C. G., Mahon, M. F., Raithby, P. R., Renouf, C. L., Richardson, C. & Stevenson, A. J. (2010). *Chem. Commun.* **46**, 5067–5069. 10.1039/c0cc00646g 20532347

[bb4] Carlucci, L., Ciani, G., Proserpio, D. M. & Visconti, M. (2011). *CrystEngComm*, **13**, 5891–5902.

[bb5] Dudek, M., Clegg, J. K., Glasson, C. R. K., Kelly, N., Gloe, K., Gloe, K., Kelling, A., Buschmann, H.-J., Jolliffe, K. A., Lindoy, L. F. & Meehan, G. V. (2011). *Cryst. Growth Des.* **11**, 1697–1704.

[bb6] Farrugia, L. J. (2012). *J. Appl. Cryst.* **45**, 849–854.

[bb7] Gans, J. & Shalloway, D. (2001). *J. Mol. Graphics Modell.* **19**, 557–559. 10.1016/s1093-3263(01)00090-0 11552684

[bb8] Groom, C. R., Bruno, I. J., Lightfoot, M. P. & Ward, S. C. (2016). *Acta Cryst.* B**72**, 171–179. 10.1107/S2052520616003954 PMC4822653 27048719

[bb9] Jayatilaka, D., Grimwood, D. J., Lee, A., Lemay, A., Russel, A. J., Taylo, C., Wolff, S. K., Chenai, C. & Whitton, A. (2005). *TONTO – A System for Computational Chemistry*. Available at: http:// hirshfeldsurface. net/

[bb10] Lamprey, H. (1960). *Ann. NY Acad. Sci.* **88**, 519–525. 10.1111/j.1749-6632.1960.tb20049.x 13758584

[bb11] McKinnon, J. J., Jayatilaka, D. & Spackman, M. A. (2007). *Chem. Commun.* pp. 3814–3816. 10.1039/b704980c 18217656

[bb12] McKinnon, J. J., Spackman, M. A. & Mitchell, A. S. (2004). *Acta Cryst.* B**60**, 627–668. 10.1107/S0108768104020300 15534375

[bb13] Sheldrick, G. M. (1996). *SADABS*. University of Göttingen, Germany.

[bb14] Sheldrick, G. M. (2008). *Acta Cryst.* A**64**, 112–122. 10.1107/S0108767307043930 18156677

[bb15] Sheldrick, G. M. (2015). *Acta Cryst.* C**71**, 3–8.

[bb16] Spackman, M. A., McKinnon, J. J. & Jayatilaka, D. (2008). *CrystEngComm*, **10**, 377–388.

[bb17] Spek, A. L. (2009). *Acta Cryst.* D**65**, 148–155. 10.1107/S090744490804362X PMC2631630 19171970

[bb18] Tan, J.-T., Zhao, W.-J., Chen, S.-P., Li, X., Lu, Y.-L., Feng, X. & Yang, X.-W. (2012). *Chem. Pap.* **66**, 47–53.

[bb19] Westrip, S. P. (2010). *J. Appl. Cryst.* **43**, 920–925.

[bb20] Wolff, S. K., Grimwood, D. J., McKinnon, J. J., Turner, M. J., Jayatilaka, D. & Spackman, M. A. (2012). *Crystal Explorer*. The University of Western Australia, Australia.

[bb21] Zhan, S.-Z., Li, M., Zhou, X.-P., Ni, J., Huang, X.-C. & Li, D. (2011). *Inorg. Chem.* **50**, 8879–8892. 10.1021/ic200854s 21863789

[bb22] Zhang, Y., Chen, B., Fronczek, F. R. & Maverick, A. W. (2008). *Inorg. Chem.* **47**, 4433–4435. 10.1021/ic800183v 18454517

